# From Cartilage to Matrix: Protocols for the Decellularization of Porcine Auricular Cartilage

**DOI:** 10.3390/bioengineering12010052

**Published:** 2025-01-09

**Authors:** Ana Caroline dos Santos, Livia Maria Barbosa de Andrade, Raí André Querino Candelária, Juliana Casanovas de Carvalho, Maria Carolina Miglino Valbão, Rodrigo da Silva Nunes Barreto, Marcelo Domingues de Faria, Rogerio Leone Buchaim, Daniela Vieira Buchaim, Maria Angelica Miglino

**Affiliations:** 1 Graduate Program in Anatomy of Domestic and Wild Animals, Faculty of Veterinary Medicine and Animal Science, University of São Paulo (FMVZ/USP), São Paulo 05508-270, Brazil; anacaroline.santos@usp.br (A.C.d.S.); liviabandrade21@gmail.com (L.M.B.d.A.); raiandre@usp.br (R.A.Q.C.); julianacasanovas@usp.br (J.C.d.C.); rodrigobarreto@usp.br (R.d.S.N.B.); rogerio@fob.usp.br (R.L.B.); danibuchaim@alumni.usp.br (D.V.B.); 2 Medical School, University of Marília (UNIMAR), Marília 17525-902, Brazil; mariacarolmv@gmail.com; 3 Department of Animal Morphology and Physiology, Faculty of Agricultural and Veterinary Sciences, São Paulo State University, Jaboticabal 14884-900, Brazil; 4 Department of Animal Anatomy, Agricultural Sciences—Federal University of Vale do São Francisco (UNIVASF), Petrolina 56300-000, Brazil; marcelo.faria@univasf.edu.br; 5 Department of Biological Sciences, Bauru School of Dentistry (FOB/USP), University of São Paulo, Bauru 17012-901, Brazil; 6 Medical School, University Center of Adamantina (FAI), Adamantina 17800-000, Brazil; 7 Postgraduate Program in Structural and Functional Interactions in Rehabilitation, Postgraduate Department, University of Marilia (UNIMAR), Marilia 17525-902, Brazil; 8 Postgraduate Program in Animal Health, Production and Environment, University of Marilia (UNIMAR), Marilia 17525-902, Brazil

**Keywords:** tissue engineering, recellularization, biological scaffolds, extracellular matrix, cartilaginous tissue, enzymatic decellularization, collagen

## Abstract

The shortage of tissues and damaged organs led to the development of tissue engineering. Biological scaffolds, created from the extracellular matrix (ECM) of organs and tissues, have emerged as a promising solution for transplants. The ECM of decellularized auricular cartilage is a potential tool for producing ideal scaffolds for the recellularization and implantation of new tissue in damaged areas. In order to be classified as an ideal scaffold, it must be acellular, preserving its proteins and physical characteristics necessary for cell adhesion. This study aimed to develop a decellularization protocol for pig ear cartilage and evaluate the integrity of the ECM. Four tests were performed using different methods and protocols, with four pig ears from which the skin and subcutaneous tissue were removed, leaving only the cartilage. The most efficient protocol was the combination of trypsin with a sodium hydroxide solution (0.2 N) and SDS (1%) without altering the ECM conformation or the collagen architecture. In conclusion, it was observed that auricular cartilage is difficult to decellularize, influenced by material size, exposure time, and the composition of the solution. Freezing and thawing did not affect the procedure. The sample thickness significantly impacted the decellularization time.

## 1. Introduction

Tissue engineering plays a central role in the creation of new tissues and organs in vitro, addressing the growing demand for transplants amid a shortage of donors [1,2]. Tissue replacement, however, is a major challenge in regenerative medicine, particularly in cases of congenital defects, trauma, or cancer surgeries [3]. The choice of biomaterials depends on the therapeutic goal (e.g., orthopedic or cardiovascular applications), and one of the biggest challenges is to ensure that these biomaterials are compatible with the physical functions of the body (mechanical compatibility) and that they do not cause adverse reactions in the immune system (biological compatibility) [4,5]. In this context, animal cartilage, particularly from bovine and porcine sources, shows promise due to its accessibility and resistance to autolysis [6,7,8,9,10]. However, using animal cartilage without sufficient decellularization can trigger unwanted inflammatory responses [11,12]. Decellularization removes donor cells, minimizing antigenicity and inflammation after transplantation while preserving the matrix structure [13,14]. This process involves physical (e.g., freezing), chemical (e.g., using detergents), and enzymatic treatments (using enzymes that degrade cellular components) tailored to the complexity of each tissue type [15,16].

Cartilage is widely used in reconstructive surgery because of its high biocompatibility and low absorption rates [17]. Researchers have explored various techniques for decellularizing auricular cartilage for both allogenic and xenogeneic transplants, as these methods result in lower immunogenic response and facilitate the rapid integration of matrix proteins [7,18,19,20,21]. External ear cartilage, which is primarily composed of type I and II collagen and elastic fibers, is dense and difficult to decellularize, but its matrix can be repopulated with specialized cells, making it suitable for transplantation or biogel production [18,22,23].

This study proposes an optimized and low-cost protocol for the decellularization of porcine auricular cartilage, a tissue particularly resistant to this type of treatment due to its density and composition. The research was conducted in response to the growing need for viable alternatives for the creation of biological scaffolds from animal tissues that are mechanically compatible and biologically safe for transplants, especially in cases where the availability of human tissues is limited. This study’s objective is to develop an efficient protocol that preserves the integrity of the extracellular matrix of auricular cartilage after decellularization, maintaining the essential properties of collagen and elastic fibers. The central hypothesis is that the combination of chemical agents, such as trypsin and SDS, associated with physical preparation methods, will result in an acellular matrix with minimal structural alteration, ready for future applications in recellularization, tissue engineering, and use in regenerative medicine.

## 2. Materials and Methods

### 2.1. Ethics Committee

This study was approved by the Ethics Committee for the use of Animals of the Faculty of Veterinary Medicine and Animal Science at the University of São Paulo (approval number: 4773231121) and the Ethics Committee for the Use of Animals of the Faculty of Agricultural and Veterinary Sciences at the São Paulo State University, Jaboticabal campus (approval number 2688/21).

### 2.2. Standardization of the Protocol for Auricular Cartilage Decellularization

#### 2.2.1. Auricular Cartilage Samples

**Figure 1:**
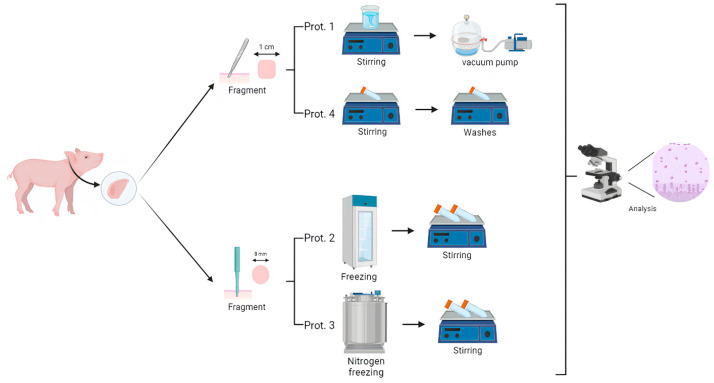
Experimental design. In Protocol 1 (Prot. 1), the dissected sample was cut into 1 cm^2^ fragments and agitated at room temperature for 10 days, followed by a vacuum pump for 2 h on days 9 and 10. Histological analyses were conducted using hematoxylin/eosin (H&E) and DAPI staining. In Protocol 2 (Prot. 2), the sample was cut into 8 mm^2^ fragments. Group A was frozen at −150 °C, while Group B was frozen at −80 °C, with both groups proceeding to agitation for 28 days. Histological analyses were also conducted using H&E and DAPI staining. In Protocol 3 (Prot. 3), the sample was again cut into 8 mm^2^ fragments, with one group frozen in liquid nitrogen and the other left unfrozen. Both groups were subjected to orbital agitation before histological analysis using H&E and DAPI staining. In Protocol 4 (Prot. 4), the samples were cut into 1 cm^2^ fragments and subjected to orbital agitation for decellularization and subsequent washings.

We obtained four pig ears from commercial slaughterhouses in Brazil. The skin and subcutaneous tissue were dissected and removed, leaving only the cartilage. Next, the cartilage was washed with distilled water and then frozen at −80 °C until decellularization (Figure 1).

#### 2.2.2. Decellularization Protocols

- Protocol 1

The samples were thawed at room temperature and cut into 1 cm fragments, ensuring both the thickest and thinnest regions of the cartilage were included. These fragments were divided into two experimental groups, each containing 12 fragments. The first group was immersed in a 1% sodium dodecyl sulfate (SDS) solution, while the second group was treated with a combined solution of 0.5% SDS and 0.5% Triton X-100. Both groups were maintained in glass containers, and the fragments were agitated at 100 rpm for 10 days, with detergent changes occurring twice daily. On the 9th and 10th days, the samples were placed in a vacuum chamber at 400 mmHg for two hours. Next, the samples were washed in 1X phosphate-buffered saline (PBS) solution under orbital agitation in three 10 min cycles and then refrigerated. Immunofluorescence staining was performed using 4′,6-diamidino-2-phenylindole (DAPI), and histological evaluation was conducted by staining the sections with hematoxylin and eosin (H&E).

- Protocol 2

The samples were washed and dissected as described in Section 2.2.1. Next, the samples were standardized in size by cutting them into uniform cartilage fragments using an 8 mm dermatological punch. The samples underwent rapid freezing and thawing cycles and were divided into two groups, each containing 42 fragments. Group A was frozen using liquid nitrogen at −150 °C for 30 min and then thawed in a water bath at 40 °C until completely thawed. Group B was previously frozen at −80 °C in a conventional freezer after dissection, as described in Section 2.2.1, and then acclimated in an ultrafreezer at −80 °C for 30 min before being thawed in a water bath at 40 °C. Each freezing and thawing cycle was repeated three times for both groups. Subsequently, each group was further divided into six treatments, with seven fragments in each subgroup, to assess differences between the freezing processes. Twelve subprotocols were tested: (a) 1% SDS solution + 1% Triton X-100 in distilled water at −150 °C; (b) 1% SDS solution + 1% Triton X-100 in distilled water at −80 °C; (c) 1% SDS solution + 1% Triton X-100 + 1% EDTA in distilled water at −150 °C; (d) 1% SDS + 1% Triton X-100 + 1% EDTA in distilled water at −80 °C; (e) 1% SDS solution + 1% Triton X-100 in 1× PBS at −150 °C; (f) 1% SDS solution + 1% Triton X-100 in 1× PBS at −80 °C; (g) 1% SDS solution in distilled water at −150 °C; (h) 1% SDS solution in distilled water at −80 °C; (i) 1% SDS solution + 1% EDTA in distilled water at −150 °C; (j) 1% SDS solution + 1% EDTA in distilled water at −80 °C; (k) 1% SDS solution in 1× PBS at −150 °C; and (l) 1% SDS solution in 1× PBS at −80 °C (Table 1).

The fragments remained immersed in the solutions under orbital agitation at 100 rpm for 28 consecutive days, with detergent changes performed three times daily. At the end of this period, the samples were washed in distilled water four times, with changes occurring every hour. Histological analyses and fluorescence microscopy (DAPI) were conducted on days 8, 17, and 28 of the decellularization process. Since the fragments were not discarded during the experiment, all 84 fragments remained available for analysis on each of these days. Therefore, 84 samples were examined on days 8, 17, and 28.

- Protocol 3

The ears were cut using an 8 mm dermatological punch, yielding 30 fragments, which were divided into two groups of 15 fragments each. In Group A, the samples were not frozen, whereas in Group B, the fragments were frozen in liquid nitrogen and then thawed at room temperature for 10 cycles. Both groups were treated with 20 mL of a 0.25% trypsin + 0.2% EDTA solution and agitated orbitally at 500 rpm at 37 °C for 16 h. After treatment, the samples were washed with distilled water four times, with a change every 30 min while agitating at room temperature. Next, a 1% SDS + 0.2N NaOH solution was applied for 36 h, with two medium changes during this period. The samples were then washed again in distilled water four times. Subsequently, a 1% SDS + Triton X-100 solution in 1× PBS was applied for 48 h, with two medium changes. Finally, the samples were washed twice in distilled water and twice in 1× PBS. At the end of each stage, one fragment was collected for histological and fluorescence microscopy (DAPI) analysis to confirm decellularization.

- Protocol 4

The ears were dissected and cut into 1 cm^2^ fragments using a scalpel. Next, 20 fragments were placed into each 50 mL Falcon tube, and 30 mL of a 0.25% trypsin + 0.2% EDTA solution was added. The tubes were kept under orbital agitation at 500 rpm and 37 °C for 14 h. After this, the samples were washed six times with distilled water, with changes occurring every 15 min at room temperature and under agitation. Then, a 1% SDS + 0.2N NaOH solution was added, and the fragments were left under agitation for 48 h, with the solution being changed four times during this period. At the end of this process, the samples were washed ten times for 15 min each to remove the alkaline solution and detergent. The fragments were then cleaned in 70% ethanol, with four 10 min baths. Finally, the samples were washed four times in distilled water and once in sterile PBS before being stored in a freezer.

### 2.3. DAPI Fluorescence

Samples were embedded in a Tissue-Tek OCT Compound and cut using a cryostat. The slides were stored in a freezer until the assay was performed. Prior to staining, the slides were thawed at room temperature for 15 min and then stained with a DAPI solution (1:10,000 in 1× PBS) in the dark for 15 min. After staining, the slides were washed with distilled water. Finally, the slides were examined and photographed under a fluorescence microscope (FV1000 Olympus IX91, Tokyo, Japan) using a blue filter to detect the remaining cell nuclei.

### 2.4. Histological Evaluation

Control and decellularized materials were processed histologically. The samples were dehydrated in a series of increasing ethanol concentrations (70, 80, 90, and 100%) for 30 min at each concentration and cleared in xylene twice for 1 h each. Next, the samples were immersed in paraffin (two baths of 1 h each) and embedded in paraffin. The resulting blocks were cut at 5 μm using a microtome, and the sections were mounted onto slides. After drying in an oven, the slides were stained with H&E, Gomori’s trichrome, Alcian blue, safranin O, and picrosirius red. Finally, the stained sections were photographed under a LEICA RM 2065 optical microscope (Leica Microsystems GmbH, Wetzlar, Germany).

### 2.5. Scanning Electron Microscopy (SEM)

The samples were fixed in Karnovsky’s solution for 48 h, followed by four washes with distilled water, each lasting 5 min, and stored in 70% ethanol. The fragments were then dehydrated and subjected to critical point drying using a Balzers CPD 020 (Balzers Union, Liechtenstein, Germany). Next, the samples were coated with a thin layer of gold using a sputtering process with an Emitech K550 (Quorum Technologies, Lewes, UK). The analysis was conducted using a scanning electron microscope (SEM Leo 435 VP, Oxford Instruments, Abingdon, UK) at the Advanced Imaging Diagnosis Center—School of Veterinary Medicine and Animal Science at the University of São Paulo (CADI-FMVZ USP).

### 2.6. Immunohistochemistry

Immunohistochemistry was used to visualize the components of native tissue and extracellular matrix and thus identify specific proteins. For this, we used the Dako kit (EnVision™ FLEX). Primary antibodies included: Collagen I (1:250; PA5-29569; Invitrogen, Bend, OR, USA), Collagen III (1:100; PA1-28870; Invitrogen), Collagen V (1:100; PA5-102416; Invitrogen), Elastin (1:250; Ab9519; Abcam, Cambridge, UK), Fibronectin (1:250; Ab2413; Abcam), and Laminin (1:500; PA1-16730; Invitrogen). The antibodies were incubated overnight in a refrigerator within a humid chamber. The sections were then developed using diaminobenzidine (DAB), prepared at a concentration of 20 μL per section (1 mL of substrate + 1 drop of chromogen). The sections were counterstained with Harris hematoxylin for 3 min, washed with distilled water, rehydrated, and photographed using a Nikon Eclipse 80i microscope (Nikon Instruments Inc., Melville, NY, USA).

### 2.7. DNA Quantification

To quantify genomic DNA (gDNA), 50 mg samples of control and decellularized cartilage were processed using the Illustra Tissue and Cells Genomic Prep Mini Spin Kit (GE Healthcare, Seoul, Republic of Korea), following the manufacturer’s instructions. The samples were digested with proteinase K and lysis buffer at 56 °C for 4 h. The purified gDNA was then analyzed using a spectrophotometer at 260 nm (Nanodrop, Thermo Scientific, Tokyo, Japan).

### 2.8. Semi-Quantification Technique for Collagen and Glycosaminoglycans

All images were semi-quantified using ImageJ win64 software (version 1.48). Ten images of different tissue regions were captured using a light microscope equipped with a 20× objective lens. Alcian blue staining, which marks glycosaminoglycans (GAGs) and acidic mucins in turquoise blue, was analyzed using manually adjusted thresholds for matrix, saturation, and brightness in ImageJ. The GAG content was quantified by calculating the average intensity of turquoise blue per tissue area. Similarly, additional images were obtained for picrosirius red staining using bright field microscopy and a circularly polarized filter, as the birefringence seen under polarized light is specific to collagen [24,25].

## 3. Results

**Figure 2:**
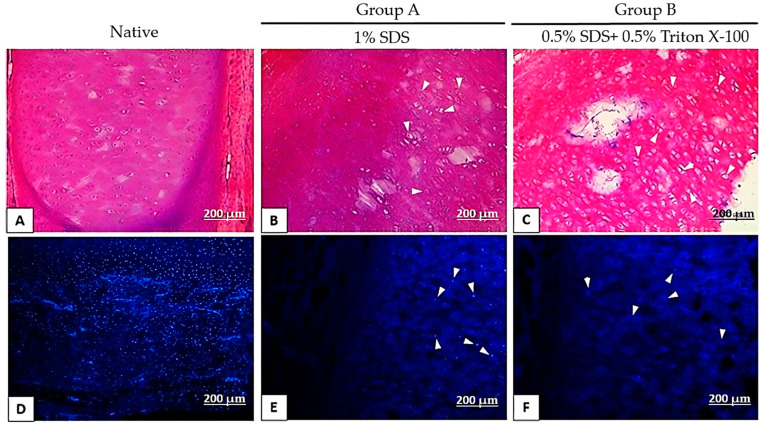
Histological analyses of porcine ears subjected to Protocol 1 after 10 days of decellularization. Group (**A**) was treated with 1% SDS. Group (**B**) was treated with 0.5% SDS + 0.5% Triton X-100. Both groups were stained with H&E and subjected to DAPI immunofluorescence. Scale bar: 200 μm. The white arrows indicate the cytoplasm and cellular nuclei highlighted by H&E staining (**B**,**C**) and DAPI immunofluorescence (**D**–**F**).

In the initial phase of the study, aimed at standardizing and stabilizing the decellularization protocol, four porcine ears were dissected, preserving only the cartilaginous tissue. Histological images of the control group show that the nuclei and cell cytoplasm are distinguishable amidst the amorphous substance and elastic fibers of the extracellular matrix in both peripheral and medial regions H&E staining revealed dark, purple-stained nuclei and pink-stained cytoplasm, facilitating cell identification (Figure 2A). Likewise, DAPI staining enabled visualization of chondrocyte nuclei (Figure 2D). The assessment of the nuclear presence, performed using H&E and DAPI staining in Protocol 1, indicated that both Protocol A (1% SDS) and Protocol B (0.5% SDS + 0.5% Triton X-100) resulted in a reduction in cell number after 10 days of decellularization compared to native tissue (Figure 2B,C). Notably, protocol B resulted in fewer cells than Protocol A (Figure 2C,F vs. Figure 2B,E). However, neither protocol demonstrated good decellularization.

**Figure 3:**
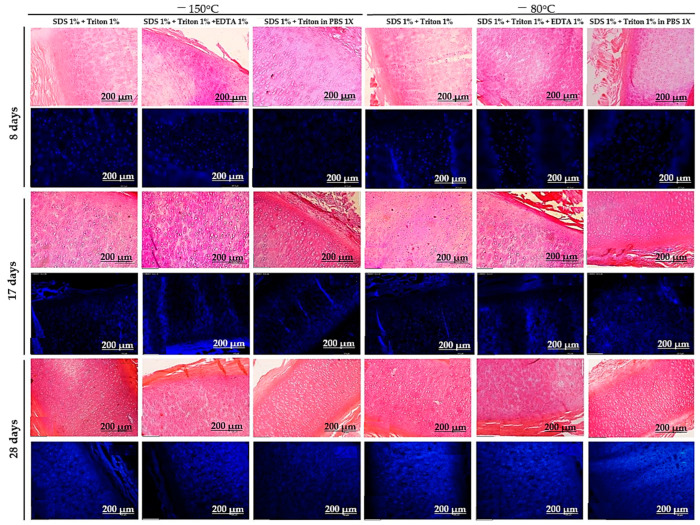
Histological sections of cartilage stained with H&E and DAPI from Pilot 2 at 8, 17, and 28 days of decellularization from protocols with the combination of two detergents. Scale bar: 200 μm.

**Figure 4:**
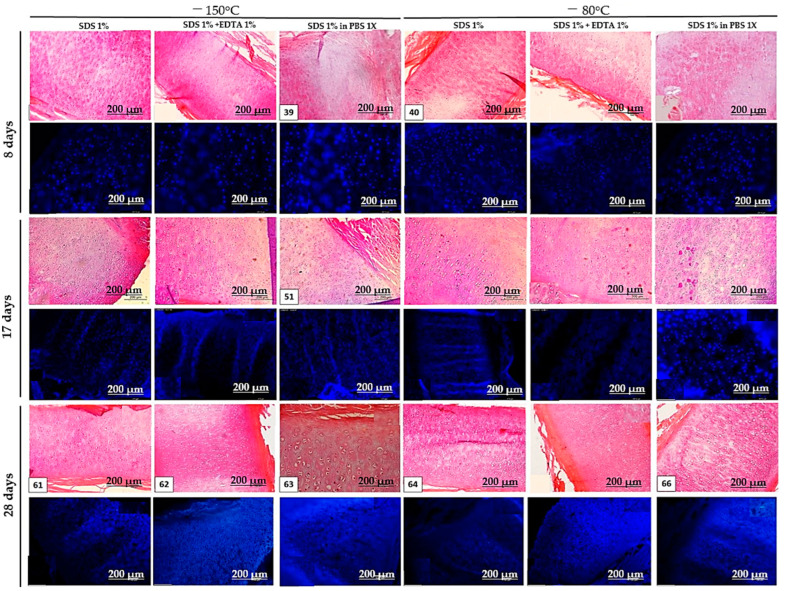
Histological sections of cartilage stained with H&E and DAPI from Pilot 2 at 8, 17, and 28 days of decellularization, using only one detergent. Scale bar: 200 μm.

A new protocol (2) was conducted, testing 12 distinct subprotocols, described in Table 2:

None of the protocols achieved satisfactory decellularization after 8 days, as cell nuclei were still evident. However, the protocols that combined two detergents (SDS and Triton X-100) showed an initial disruption of matrix fibers, which enhanced detergent penetration and facilitated cell lysis and removal. By 17 days, the peripheral regions of the tissue were the first to decellularize, with thinner fragments decellularizing faster than thicker ones. This pattern was consistent across all protocols but was most pronounced in the 1% SDS + 1% Triton X-100 in 1× PBS (−80 °C and −150 °C) protocols. No significant differences were observed between the freezing temperatures of −80 °C and −150 °C. After 28 days, the best results were seen in the 1% SDS + 1% Triton X-100 in 1× PBS (−150 °C) and 1% SDS + 1% Triton X-100 in 1× PBS (−80 °C) protocols (Figure 3 and Figure 4). Despite these improvements, the tissue was still incompletely decellularized.

**Figure 5:**
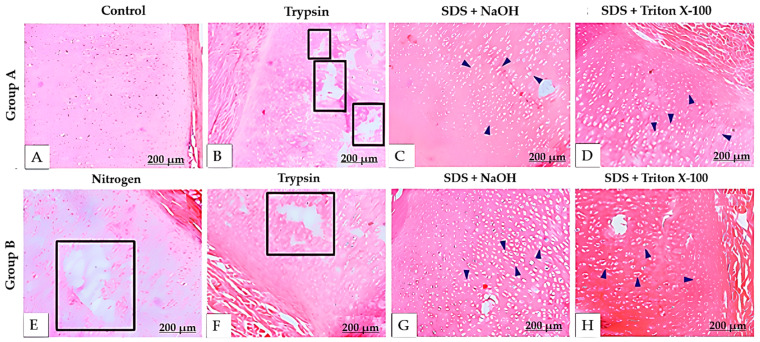
Histological sections of decellularized porcine auricular cartilage tissue stained with hematoxylin and eosin (H&E) in Protocol 3. Scale bar: 200 μm. Group A (**A**–**D**) was not subjected to liquid nitrogen freezing, whereas Group B was (**E**–**H**). Blue arrows indicate areas devoid of chondrocytes, and black squares indicate damage to the extracellular matrix architecture caused by nitrogen and trypsin.

Given the unsatisfactory results from Protocols 1 and 2, we developed Protocol 3, which involved dividing the samples into two groups. Group A samples were not subjected to liquid nitrogen freezing, whereas Group B underwent this process. The remaining steps were identical for both groups: treatment with trypsin for 16 h, followed by 1% SDS + 0.02 N NaOH for 36 h, and finally 1% SDS + 1% Triton X-100 in 1× PBS for 48 h. This protocol successfully achieved full decellularization of the samples (Figure 5). H&E staining showed the presence of elastic fibers and the absence of chondrocytes, with both their cytoplasm and nuclei removed, resulting in empty lacunae within the tissue. However, trypsin substantially changed the extracellular matrix architecture, likely due to prolonged enzyme exposure. Liquid nitrogen freezing also damaged the extracellular matrix.

**Figure 6:**
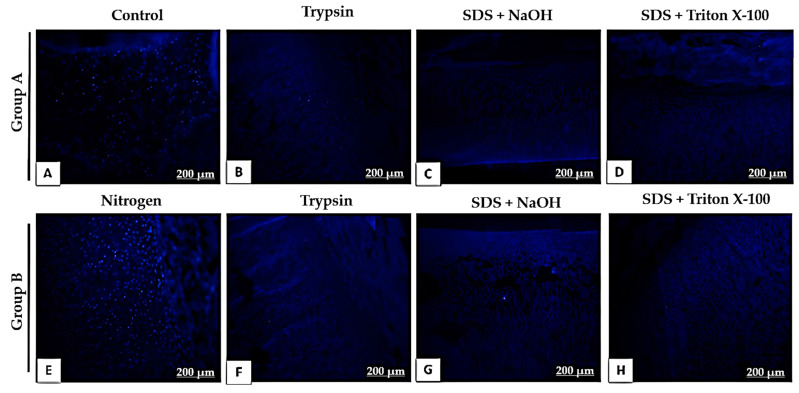
DAPI fluorescence imaging of decellularized porcine auricular cartilage tissue from Protocol 3. Group A (**A**–**D**) was not subjected to liquid nitrogen freezing, whereas Group B was (**E**–**H**). Scale bar: 200 μm.

**Figure 7:**
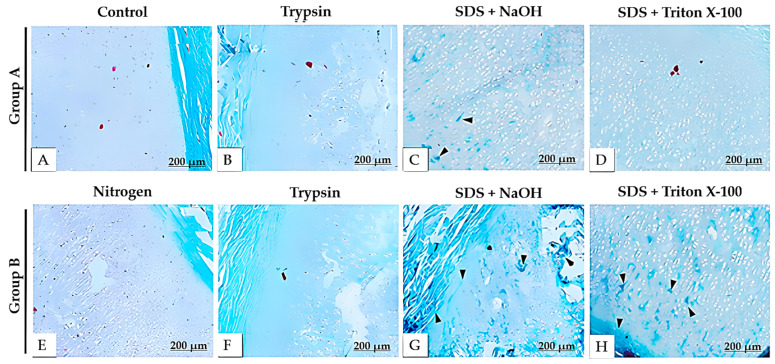
Histological sections of decellularized porcine auricular cartilage tissue stained with Gomori’s trichrome from Protocol 3. Group A (**A**–**D**) was not subjected to liquid nitrogen freezing, whereas Group B was (**E**–**H**). Scale bar: 200 μm. Black arrows highlight areas with high collagen abundance, even after the decellularization process.

DAPI staining (Figure 6) confirmed the successful removal of cells by the 5th day, showing a significant reduction compared to the native tissue. This result indicates that trypsin effectively accelerated the decellularization process. Moreover, the alkaline solution contributed to the effective decellularization of the cartilaginous tissues. Gomori’s trichrome staining (Figure 7) revealed that the alkaline solution did not change the histological properties of the collagen fibers. Despite the decellularization process, a considerable amount of collagen was preserved, as evidenced by the increased intensity of the blue tones.

**Figure 8:**
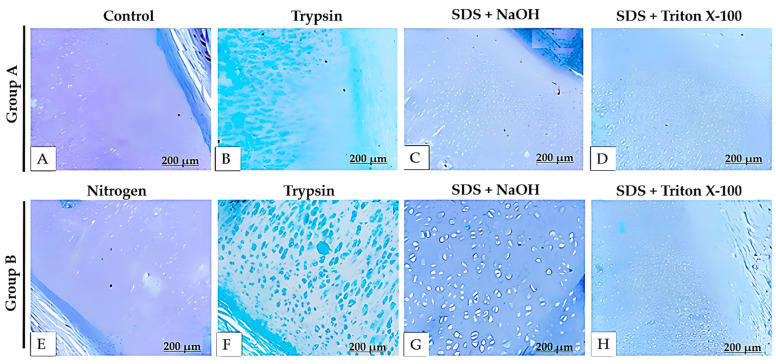
Histological sections of decellularized porcine auricular cartilage tissue stained with Alcian Blue from Protocol 3. Group A (**A**–**D**) was not subjected to liquid nitrogen freezing, whereas Group B was (**E**–**H**). Scale bar: 200 μm.

**Figure 9:**
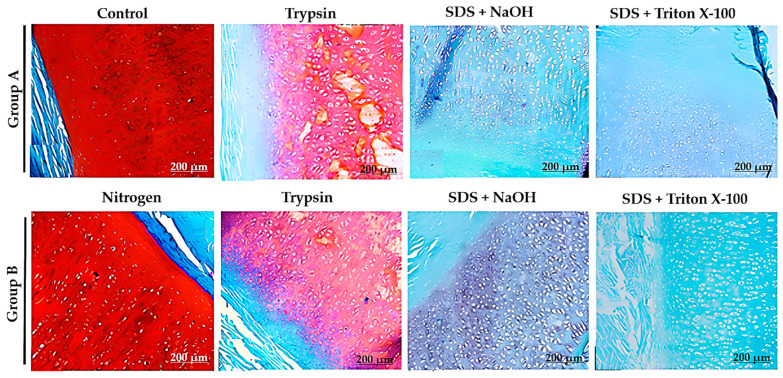
Histological sections of decellularized porcine auricular cartilaginous tissue stained with safranin O from Protocol 3. Scale bar: 200 μm.

The Alcian blue (Figure 8) and safranin O (Figure 9) staining allowed us to observe the presence of glycosaminoglycans (GAGs). However, there was a significant decrease in the quantity of these compounds in the decellularized samples, as indicated by the reduced intensity of blue (Alcian blue) and red (safranin O) staining.

**Figure 10:**
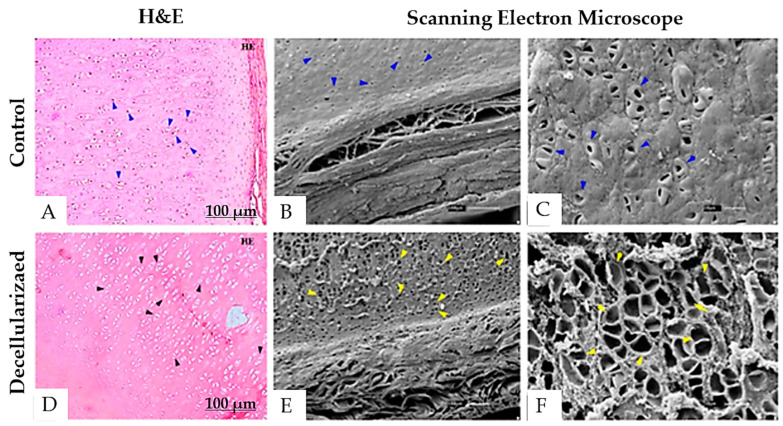
Histological analysis and scanning electron microscopy of porcine auricular cartilage samples submitted to Protocol 4. Scale bar: 100 μm. (**A**–**C**) Control group. (**D**–**F**) Decellularized tissue. Blue arrows indicate the presence of nuclei in the control group and intact cellular lacunae, whereas black arrows highlight the absence of nuclei after decellularization, whereas yellow arrows highlight empty lacunae and the absence of cell bodies.

To accelerate the decellularization time, we developed Protocol 4 building on the findings from Protocol 3, which indicated that the SDS + NaOH solution facilitated complete decellularization. Protocol 4 yielded even better results, reducing the decellularization time to just 3 days. Analysis of the H&E-stained slides and Scanning electron microscopy (Figure 10) confirmed complete decellularization, as evidenced by the presence of empty lacunae and the absence of cell bodies. Scanning electron microscopy revealed morphological changes before and after treatment. The decellularized group showed higher retention of collagen and elastic fibers, which appeared densely intertwined within the matrix, forming a more uniform collagen network compared to the thinner native cartilage. Additionally, the empty lacunar structures observed in the H&E staining were also present, and the cartilage surfaces appeared to remain intact after decellularization.

**Figure 11:**
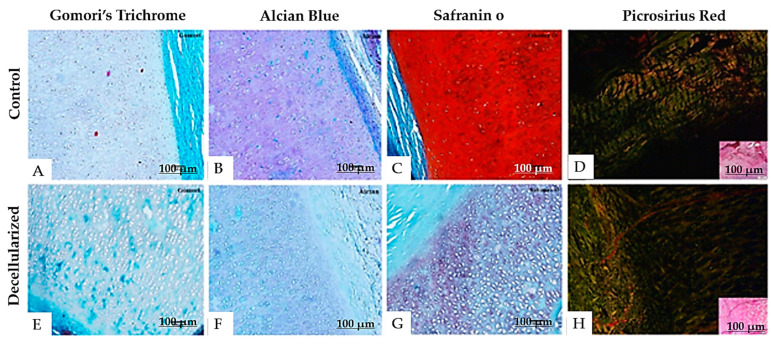
Photomicrograph of porcine auricular cartilage stained with Gomori’s trichrome, alcian blue, safranin O and picrosirius red (Protocol 4). Scale bar: 100 μm. Decellularized group (**E**–**H**) compared to the control group (**A**–**D**).

Gomori’s trichrome and picrosirius red staining (Figure 11A,D,E,H) demonstrated the alignment of collagen fibers and a high abundance of collagen, even after decellularization. No significant differences in collagen structure or distribution were noted between the control and decellularized groups, indicating that the protocol effectively maintained the integrity of the extracellular matrix.

A reduction in extracellular matrix components was evident in the decellularized cartilage samples, as evidenced by the decrease in turquoise blue in Alcian blue staining and red in safranin O staining (Figure 11B,C,F,G).

**Figure 12:**
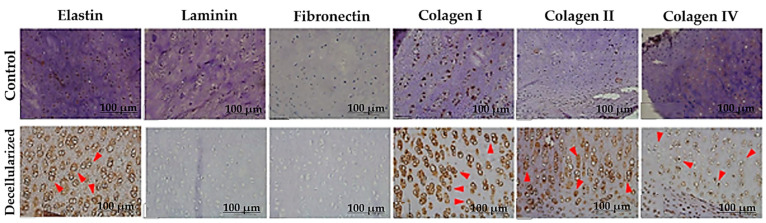
Immunohistochemical analysis of porcine auricular cartilage stained for elastin, laminin, fibronectin, collagen I, collagen III, and collagen V. Red arrows indicate regions with positive expression of the matrix proteins elastin, collagen I, collagen III, and collagen V. Scale bar: 100 μm.

Immunohistochemical analysis revealed positive expression of structural proteins (collagen I, collagen III, collagen V, and elastin) as well as adhesive glycoproteins (fibronectin and laminin) in the control tissue (Figure 12). However, fibronectin and laminin showed only isolated staining points (Figure 12). In the decellularized auricular cartilage, structural proteins were positively expressed in both the cartilage and perichondrium, with more intense staining compared to the native tissue, indicating their preservation during the decellularization protocol. However, laminin and fibronectin were not detected in the decellularized samples, suggesting their removal during decellularization.

**Figure 13:**
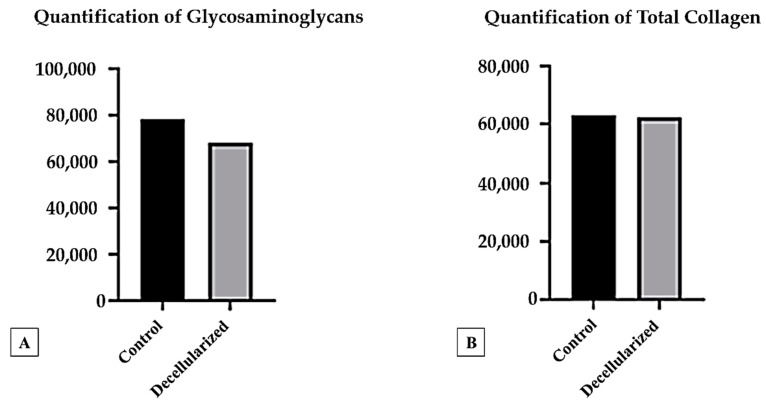
Quantification of glycosaminoglycans (GAGs) (**A**) and quantification of total collagen (**B**) in native (control) and decellularized porcine auricular cartilage tissue. Results are expressed as micrograms per milligram of tissue (µg/mg).

We analyzed the gDNA content per milligram of tissue to evaluate the efficacy of the decellularization process in native (control) and decellularized materials. The gDNA content in the decellularized samples was 13.05 ng/mg, below the established threshold of 50 ng/mg, whereas the control sample measured 212.7 ng/mg. Protocol 4 exhibited superior results, although the decellularized samples contained fewer glycosaminoglycans (GAGs) than the native (control) tissue, indicating a reduced preservation of this component (Figure 13A). The total collagen concentration was not significantly affected by the decellularization process (Figure 13B).

## 4. Discussion

This work addresses the optimization of a low-cost protocol for the decellularization of porcine auricular cartilage, aiming to meet the growing demand for viable alternatives in the creation of biological scaffolds from animal sources. Tissue engineering seeks to replace damaged tissues and meet the demand for transplants, which is limited due to the shortage of human donors. Animal cartilage, especially porcine, shows promise for transplants due to its resistance to autolysis and accessibility, but it requires decellularization to minimize unwanted immune reactions. The goal of this study is to develop an effective protocol that preserves the integrity of the extracellular matrix while maintaining the properties of collagen and elastic fibers. It was observed that the protocol using 0.25% trypsin and 1% SDS + 0.2 N NaOH (Protocol 4) is the most efficient in removing cells while preserving the fibers, although with a slight loss of GAGs. The research concludes that fragment size and the use of trypsin and NaOH have a greater impact on the success of the process than prior freezing, offering a promising solution for the development of safe and mechanically compatible scaffolds for use in regenerative medicine.

The choice of fragment size varied depending on the specific goals of each protocol. In Protocols 1 and 4, 1 cm fragments were used to encompass both thick and thin regions of the cartilage, ensuring a comprehensive evaluation of the decellularization process across different tissue depths. Conversely, Protocols 2 and 3 employed 8 mm fragments to standardize the samples and facilitate rapid freezing and thawing cycles, promoting uniform exposure to the decellularization agents. This differentiation optimized the efficiency and consistency of the process, depending on the methodological requirements.

Although cartilage is rigid and flexible, it lacks the regenerative capacity of other self-repairing tissues (e.g., bone), making post-injury healing particularly difficult. This limitation creates a demand for matrices suitable for cartilage replacement or repair [26]. In this context, tissue engineering has emerged as a promising solution, with biomaterials such as scaffolds showing exceptional results [27,28]. The characterization of an appropriate scaffold or biomaterial involves several parameters, such as biocompatibility, mechanical stability, and availability [29]. Additionally, the developed matrices must provide a niche conducive to cell proliferation, adhesion, and migration, as well as promote the synthesis of newly produced extracellular matrix components. Therefore, it is essential to have an adequate procedure that enables the elimination of immunogenic components, minimizes the risk of rejection and disease transmission, and preserves the integrity of the extracellular matrix [14,30]. Based on these considerations, the present study evaluated the efficacy of various decellularization protocols for porcine auricular cartilage, analyzing the biomechanical and structural changes following decellularization.

Cartilage is widely used in rhinoplasty and reconstructive surgeries due to its low absorption rate and high biocompatibility [17,31]. Auricular cartilage, in particular, has shown high tolerance when treated with detergents and alkaline solutions, even after being injected into paralyzed canine vocal folds [32]. Various techniques have been explored for the decellularization of ear cartilage for use in allogenic and xenogeneic transplants, given that its matrix proteins elicit an immunogenic response and facilitate rapid integration with host tissues [18,19,20,33,34]. For these reasons, this biomaterial was chosen for the present study.

Histologically, elastic cartilage is characterized by the presence of elastic components that stain for chondrocytes, amorphous ground substances, as well as collagen and elastic fibers, all of which contribute to the preservation of cartilage integrity [35,36]. These features enabled us to determine whether the decellularization of the material was achieved. The efficiency of a given decellularization protocol depends on the tissue of interest [37,38]. When different chemical agents are used—such as ionic and non-ionic detergents, alkaline solutions, and enzymes—the success of the process depends on the quality of the post-decellularized tissue, as indicated by the remaining elements of the extracellular matrix.

In the first decellularization protocol, we divided the material into two groups: one treated with SDS and the other with a combination of SDS and Triton X-100. In both groups, the cellular nuclei were not fully removed, and the extracellular matrix remained intact. These findings differ from those of Lou et al. [39], who successfully decellularized porcine auricular cartilage using the same detergents. This discrepancy may be explained by differences in sample thickness, as those authors used 10 mm thick sections, which likely facilitated more efficient detergent penetration.

Detergents such as SDS and Triton X-100 are commonly used for tissue decellularization [40,41,42]. Previous studies have applied these detergents to remove cells from various tissues, including tracheal cartilage [43], auricular cartilage [39], and nasal septal cartilage [44]. For this reason, we continued to use these detergents in Protocol 2 while testing twelve subprotocols that combined physical and chemical methods, as well as varying concentrations and processing times. However, staining with H&E and DAPI labeling revealed that cellular remnants persisted in the samples even after 28 days of processing.

We found that the peripheral regions of the ear were the first to achieve complete decellularization. This finding contrasts with the results of 23. Ferreira et al. [23], who reported that the central region decellularized first but aligns with the observations of Hong [45]. Xu et al. [46] observed different decellularization thresholds according to the zones of the involved tissue, specifically contrasting the central and peripheral regions at the tendon-bone interface.

These authors used decellularization protocols with chemical detergents (SDS and Triton X-100) at different concentrations and time points (48 and 72 h), along with physical methods such as freeze–thaw cycles, ultrasound, perfusion, and hydrostatic washing. Their experiment was conducted on porcine calcaneal tendons measuring 2 mm thick, 30 mm long, and 5 mm wide, and the calcified regions consisted of dense connective tissue, non-calcified fibrocartilage, calcified fibrocartilage, and bone tissue. They found that the fibrocartilage regions exhibited a higher cell density. Thus, the type of tissue, decellularization methods, and duration of detergent exposure are critical factors influencing the outcomes of the decellularization process.

Gilbert et al. [47] define effective decellularization as the complete removal of cellular material (including the plasma membrane, cytoplasm, organelles, and cell nuclei) while preserving the composition, biological activity, and integrity of the remaining extracellular matrix. Considering the prolonged decellularization period and the results observed, we developed Protocol 3 to accelerate the process, using enzymatic methods and alkaline solutions as described by Ferreira [48], Ferreira [23], and Araújo [49].

Among the chemical decellularization methods, alkaline solutions (e.g., ammonia, sodium sulfide, and sodium hydroxide) are widely used because their pH helps solubilize cellular components, including plasma membranes, organelles, and nucleic acids [37]. Our histological analyses indicate that Protocol 3 achieved complete decellularization of the samples within 5 days. Additionally, Protocol 3 preserved collagen fibers, although a significant reduction in glycosaminoglycans was observed, a result that was anticipated and previously reported by other authors [24,25].

The use of alkaline solutions for decellularization is one of the most effective approaches, as our results show the lysis of chondrocytes while preserving extracellular matrix fibers. This method has also yielded satisfactory outcomes in other tissues, including the pericardium, liver, tendon, and auricular cartilage, and has shown better biocompatibility compared to methods that use detergents and 98% glycerin [37,50].

To achieve the best protocol in the shortest timeframe and at a low cost, we refined the results of the previous pilot protocol to enhance the preservation of extracellular matrix components and facilitate future research in the field. Thus, in Protocol 4, the samples were neither frozen nor thawed, the exposure time to trypsin was reduced, and the detergent application was accelerated. The efficiency of the decellularizing agents was evaluated using scanning electron microscopy, histological techniques, immunohistochemistry, and quantification of collagen and glycosaminoglycans.

An ideal protocol should remove cellular components from the tissue, as indicated by gDNA quantification (<50 ng of DNA) while preserving the structure and components of the extracellular matrix [24,51]. Protocol 4 met these criteria for complete decellularization of auricular cartilage, reducing the gDNA content to 13.05 ng/mg of tissue. This reduction minimizes the likelihood of immune responses in “in vivo” transplants.

Despite the loss of cells, preserving the structural integrity of the matrix is equally important. Collagen, a primary structural protein in the cartilage extracellular matrix, provides mechanical strength and regulates chondrogenic differentiation [52]. Along with elastin, these macromolecules play important functional and structural roles by providing tensile strength and connecting cell surface receptors with glycosaminoglycans [53,54]. Preserving collagen and elastic fibers during decellularization is an important challenge. Our findings (Protocol 4) demonstrate that these fibers remained compact, retained their proper orientation, and showed no drastic changes after the decellularization process. Histological analysis, using sections stained with picrosirius red and Gomori’s trichrome, confirmed these results.

Immunohistochemical evaluations, specifically the immunostaining of decellularized tissue, demonstrated positive expression compared to control tissues. The expression and organization of collagen fibers indicated that the cellular removal processes preserved extracellular matrix proteins, consistent with findings in previous studies [24,25,55,56]. Additionally, scanning electron microscopy images supported these findings, showing that the alignment of collagen and elastic fibers remained unchanged in the decellularized samples.

The quantification of collagen showed minimal changes in the decellularized groups compared to the native matrix, with non-significant differences in collagen concentrations. However, proteoglycans—other vital proteins of the cartilage matrix—were reduced after decellularization. The removal of GAGs is crucial, as it promotes cellular loss, regulates water flow, and facilitates the diffusion of decellularizing agents throughout the matrix [57]. Staining results using Alcian blue and safranin O demonstrated both qualitative and quantitative loss of GAGs, consistent with findings reported by Changchen et al. [25] and Zaid et al. [24].

This study presents limitations that should be considered, such as the partial loss of GAGs in the decellularization protocol, which may affect the functionality of the extracellular matrix. Although the results are within expectations regarding the preservation of collagen and elastic fibers, further testing is needed to assess the recellularization capacity and tissue integration in more complex biological models. Future perspectives include improving the protocol to minimize the loss of structural components and conducting in vivo studies to validate the safety and efficacy of the scaffolds under physiological conditions. It is essential to ensure that the material is safe and biologically compatible and that the decellularization process can be scaled up with strict quality control, meeting regulatory requirements for human use.

## 5. Conclusions

Auricular cartilage is characterized by a dense structure with a high concentration of elastic fibers, which complicates the decellularization process. We found that standardizing the size of the fragments to be decellularized has proven to be an effective strategy. Our analyses indicate that the decellularization protocol using 0.25% trypsin, 1% SDS + NaOH 0.2 N (Protocol 4) is the most effective. This protocol rapidly and efficiently removes chondrocytes from the tissues while preserving the elastic and collagen fibers. Although there is some loss of GAGs, this protocol causes no significant changes in the conformation of the extracellular matrix. Contrary to the assertions of various authors, we found that freezing and thawing the samples before decellularizing elastic cartilage appears to have little direct impact on the process. Instead, the outcomes are more influenced by fragment size, the action of trypsin, and the NaOH solution.

## Figures and Tables

**Table 1:** Overview of decellularization protocols and subprotocols.

Protocol	Subprotocols
**Protocol 1**	- Group 1: 1% SDS solution
	- Group 2: 0.5% SDS + 0.5% Triton X-100 solution
**Protocol 2**	Two Detergents:
	- 1% SDS + 1% Triton X-100 in distilled water at −150 °C
	- 1% SDS + 1% Triton X-100 in distilled water at −80 °C
	- 1% SDS + 1% Triton X-100 + 1% EDTA in distilled water at −150 °C
	- 1% SDS + 1% Triton X-100 + 1% EDTA in distilled water at −80 °C
	- 1% SDS + 1% Triton X-100 in 1× PBS at −150 °C
	- 1% SDS + 1% Triton X-100 in 1× PBS at −80 °C
	One Detergent:
	- 1% SDS in distilled water at −150 °C
	- 1% SDS in distilled water at −80 °C
	- 1% SDS + 1% EDTA in distilled water at −150 °C
	- 1% SDS + 1% EDTA in distilled water at −80 °C
	- 1% SDS in 1× PBS at −150 °C
	- 1% SDS in 1× PBS at −80 °C
**Protocol 3**	- Group A (No Freezing): 0.25% trypsin + 0.2% EDTA solution for 16 h, followed by 1% SDS + 0.2N NaOH for 36 h, then 1% SDS + Triton X-100 in 1× PBS for 48 h.
	- Group B (Frozen and Thawed): 0.25% trypsin + 0.2% EDTA solution for 16 h, followed by 1% SDS + 0.2N NaOH for 36 h, then 1% SDS + Triton X-100 in 1× PBS for 48 h.
**Protocol 4**	- Trypsin + EDTA for 14 h
	- 1% SDS + 0.2N NaOH for 48 h
	- 1% SDS + Triton X-100 in 1× PBS for 48 h

**Table 2:** Division of Protocol 2 based on the use of one and two detergents.

Use of Two Detergents	SDS 1% + Triton X-100 1% (−150 °C)	SDS 1% + Triton X-100 1% (−80 °C)	SDS 1% + Triton X-100 1% + EDTA 1% (−150 °C)	SDS 1% + Triton X-100 1% + EDTA 1% (−80 °C)	SDS 1% + Triton X-100 1% in 1× PBS (−150 °C)	SDS 1% + Triton X-100 1% in 1× PBS (−80 °C)
Use of One Detergent	SDS 1% (−150 °C)	SDS 1% (−80 °C)	SDS 1% + EDTA 1% (−150 °C)	SDS 1% + EDTA 1% (−80 °C)	SDS 1% in 1× PBS (−150 °C)	SDS 1% in 1× PBS (−80 °C)

## Data Availability

Data are contained within the article.
